# Shift in the B cell subsets between children with type 1 diabetes and/or celiac disease

**DOI:** 10.1093/cei/uxad136

**Published:** 2023-12-22

**Authors:** Andrea Tompa, Maria Faresjö

**Affiliations:** Department of Natural Science and Biomedicine, School of Health and Welfare, Jönköping University, Jönköping, Sweden; Division of Diagnostics, Region Jönköping County, Jönköping, Sweden; Department of Natural Science and Biomedicine, School of Health and Welfare, Jönköping University, Jönköping, Sweden; Department of Life Sciences, Division of Systems and Synthetic Biology, Chalmers University of Technology, Gothenburg, Sweden

**Keywords:** B cell subsets, type 1 diabetes, celiac disease, children, flow cytometry

## Abstract

Our purpose was to characterize the pattern of B cell subsets in children with a combined diagnosis of type 1 diabetes (T1D) and celiac disease (C) since children with single or double diagnosis of these autoimmune diseases may differ in peripheral B cell subset phenotype patterns. B cells were analyzed with flow cytometry for the expression of differentiation/maturation markers to identify transitional, naive, and memory B cells. Transitional (CD24^hi^CD38^hi^CD19^+^) and memory Bregs (mBregs; CD24^hi^CD27^+^CD19^+^, CD1d^+^CD27^+^CD19^+^, and CD5^+^CD1d^+^CD19^+^) were classified as B cells with regulatory capacity. Children with a combined diagnosis of T1D and C showed a pattern of diminished peripheral B cell subsets. The B cells compartment in children with combined diagnosis had higher percentages of memory B subsets and Bregs, including activated subsets, compared to children with either T1D or C. Children with combined diagnosis had a lower percentage of naive B cells (CD27^−^CD19^+^; IgD^+^CD19^+^) and an increased percentage of memory B cells (CD27^+^CD19^+^; IgD^−^CD19^+^). A similar alteration was seen among the CD39^+^ expressing naive and memory B cells. Memory Bregs (CD1d^+^CD27^+^CD19^+^) were more frequent, contrary to the lower percentage of CD5^+^ transitional Bregs in children with a combined diagnosis. In children with either T1D or C, the peripheral B cell compartment was dominated by naive cells. Differences in the pattern of heterogeneous peripheral B cell repertoire subsets reflect a shifting in the B cell compartment between children with T1D and/or C. This is an immunological challenge of impact on the pathophysiology of these autoimmune diseases.

## Teaser

A larger compartment of naive B and Breg subsets, including activated subsets, and smaller compartment of the memory B and Breg subsets are seen in children with isolated type 1 diabetes (T1D) or celiac disease (C). In children with the combined diagnosis, a shifting of the naive B subset compartment toward a more mature/differentiated B cell compartment was observed, indicating another type of disturbance of the B cell mediated immune regulation compared to what is present in the context of only one isolated autoimmune disease. Differences in the pattern of heterogeneous peripheral B cell repertoire subsets reflect a shifting in the B cell compartment between children with T1D and/or C.

## Introduction

B lymphocytes (B cells) are complex modulators of immune responses, including both activating and inhibitory roles [1]. Evidence indicates several roles for B cells in autoimmune diseases *via* multiple mechanisms by secreting autoantibodies, presenting antigens as well as providing co-stimulation and cytokine secretion [2]. The heterogeneity of peripheral B cells and B cell subsets reflects their respective roles in regulatory/effector functions and contradictive participation in both protective and pathogenic responses [3]. Different surface markers can identify canonical phenotypes (transitional, naive, and memory B cells); transitional B cells express CD24, CD38, and CD10, whereas CD27 and IgD are widely used to separate B cells into naive and memory subsets [4–7]. In the context of several autoimmune diseases, naive B cells may contribute to prevent the inflammatory response, whereas memory B cells primarily act to resolve active disease exacerbation [2, 8].

Regulatory B cells (Bregs) originate from various B cell subsets and B cells at different development stages [9–12]. Similar, to Tregs, these Breg cells maintain self-tolerance and contribute to preventing autoimmunity [1, 2]. Available evidence indicates that the ‘most efficient’ IL-10-producing B cells are found within the CD24^high(hi)^CD38^hi^ transitional B cell subsets and CD24^hi^CD27^+^ B cells, also known as transitional Breg (tBreg) cells and memory Breg (mBreg) cells, respectively [9]. These B cells can suppress CD4^+^ T-cell activation or proliferation as well as inhibit the differentiation of naive CD4^+^ T cells into T helper (Th) 1 or Th17 cells [9, 13–15]. Subsets of peripheral B cells have furthermore been described as the phenotypes CD24^hi^CD38^hi^CD5^+^CD1d^hi/+^, CD24^+^CD38^hi^CD5^+^CD1d^+^IgD^−^, and CD27^−^CD24^hi^CD38^hi^ with regulatory capacity [10, 11, 14]. Bregs expressing CD1d and CD5 suppress proliferation and differentiation of Th1/Th17 immune responses, and defects in CD1d^hi^CD5^+^ Bregs can exacerbate disease symptoms of autoimmune diseases in murine models [16–18].

There is evidence that Bregs also regulate immunity via surface molecules, such as CD25 [19], CD39 [20, 21], CD95 [19], and chemokine receptor 7 (CCR7) [22]. B cells expressing CD25, the alpha chain of the IL-2 receptor, are able to co-stimulate or downregulate T-cell responses but can also induce apoptosis in a subpopulation of activated T cells [13, 19]. T-cell suppression by activated B cells is associated with the upregulation of CD39 on the surface of B cells [21]. Activated CD39^+^ Bregs represent enzymatically active suppressor cells that produce adenosine-59-monophosphate and adenosine and use the adenosinergic pathway to mediate suppression of effector T cells. Apoptosis-associated extracellular CD95 is rapidly upregulated upon B cell activation, and alterations in CD95^+^ B cell subsets have been observed in autoimmune disorders [23, 24]. Chemokine receptor 7 contributes to the trafficking of B cells and activated dendritic cells to and within secondary lymphoid tissue, suggesting a role as an important regulator of different immune responses [25].

Alterations in the number of different circulating Breg subsets and impairment in the ability to secrete IL-10 have been shown in autoimmune diseases (e.g. systemic lupus erythematosus, rheumatoid arthritis, and multiple sclerosis) [9, 11, 18]. Bregs can either prevent the onset of disease [26] or delay the onset of diabetes via B cell depletion, indicating the dual pathological role of B cells [27]. In patients with type 1 diabetes (T1D), altered numbers of IL-10-secreting B cells in peripheral blood have been shown compared to healthy controls [28]. Celiac disease (C), also considered an autoimmune disease, includes an increased percentage of CD24^hi^CD38^hi^ Bregs [29], as well as increased expression of CD1d on IL-10 secreting B cells under chronic intestinal inflammatory conditions [30].

Taken together, B cells are complex modulators in autoimmunity able to co-stimulate or downregulate T-cell responses. Based on existing evidence, the activation status of the immune responses might be reflected in their phenotypical pattern and hence their pathophysiological mechanisms on different autoimmune diseases. To our knowledge, no prior studies have examined the phenotypical pattern of B cell subsets in children with a combination of T1D and C. This study thus aimed to acquire more knowledge of the phenotypical pattern of peripheral B cell subsets in children with a combination of T1D and C in an *ex vivo* context.

## Material and methods

### Study population

The cohort includes samples collected from a total of 36 children divided into four study groups: those diagnosed with T1D (*n* = 9), C (*n* = 9), both diagnoses (*n* = 9), and a reference group consisting of children without any of these diseases (*n* = 9). Characteristics (sex, age, and duration of diseases) of the different study groups are presented in Table 1. Children in the different groups were age- and gender-matched as far as possible.

**Table 1.** Characteristics of the study groups T1D + C, T1D, C, and Ref

	T1D + C	T1D	C	Ref
*n*	9	9	9	9
Sex: F (*n*)/M (*n*)	6/3	5/4	5/4	6/3
e(years)	10.5 (5.0/6.2)	10.0 (3.3/6.0)	10.0 (2.5/7.0)	11.0 (3.3/5.7)
Female	10.8 (4.9/5.7)	10.0 (1.6/2.6)	10.0 (4.8/7.0)	11.2 (2.5/3.9)
Male	10.5 (–/5.8)	10.0 (5.5/6.0)	10.0 (1.9/2.5)	8.0 (−/4.4)
Duration T1D (years)	3.6 (3.1/9.6)	5.4 (6.2/9.8)	–	–
Female	3.6 (2.8/3.6)	5.4 (5.9/8.0)	–	–
Male	3.6 (–/9.6)	3.8 (8.2/9.8)	–	–
Duration C (years)	1.9 (4.0/9.7)	–	5.8 (7.1/11.0)	–
Female	3.3 (5.5/9.7)	–	7.1 (7.0/−11.0)	–
Male	0.8 (–/1.9)	–	2.6 (4.4/4.8)	–

Age and disease duration presented as median (IQR/range).

Abbreviations: F, females; M, males; T1D + C, children with combined type 1 diabetes and celiac disease; T1D, children with type 1 diabetes; C, children with celiac disease; Ref, reference children.

The general criteria for inclusion in the study were that children showed no signs of colds or other infections at the time of sample collection. Further, neither the reference nor their first-degree relatives displayed any signs of T1D, C, or other autoimmune diseases, based on a self-reported questionnaire.

T1D was diagnosed according to the International Society for Pediatric and Adolescent Diabetes guidelines, i.e. symptoms of diabetes plus casual plasma glucose concentration ≥ 11.1 mmol/l (200 mg/dl) or fasting plasma glucose ≥ 7.0 mmol/l (≥126 mg/dl) or 2-hour post-load glucose ≥ 11.1 mmol/l (≥200 mg/dl) during an oral glucose tolerance test. The duration of T1D was defined as the time between the date of diagnosis and the study sample collection date (Table 1). Celiac disease was diagnosed according to the modified version of The European Society of Pediatric Gastroenterology and Nutrition criteria. The duration of C was defined as the time from the date of biopsy-confirmed diagnosis until the date of sample collection (Table 1). All children in the double-diagnosis group were diagnosed with T1D before being diagnosed with C.

The study was reviewed and approved by the Research Ethics Committee of the Faculty of Health Sciences, Linköping University, Linköping, Sweden and the Regional Ethics Committee for Human Research, Linköping (approval number: Dr M89-2006 and complementary Dr: 2012/27-32).

In agreement with the Declaration of Helsinki, information was given both orally and written to all participants and their parents or responsible guardians. All children received comprehensible information adapted for their age. Informed consent was obtained from the children’s guardians.

### Sample collection and isolation of PBMC

Flow cytometric phenotyping of B cell subsets was performed on the 36 cryopreserved peripheral blood mononuclear cell (PBMC) samples, isolated from 20 mL sodium-heparinized venous blood samples using Ficoll-Paque density gradient centrifugation (Pharmacia Biotech, Sollentuna, Sweden), from children with T1D and/or C and reference children. PBMC were after isolation washed with phosphate-buffered saline (PBS) three times, and finally resuspended in cell medium [31, 32].

### Cryopreservation and thawing of PBMC

For cryopreservation, 4°C freezing media (consisting of 10% dimethyl sulfoxide [Sigma-Aldrich AB], 40% FCS, and 50% RPMI-1640) were added to cell suspension dropwise under continuous mixing. One milliliter aliquots, i.e. 5 × 10^6^ PBMC/mL in cryotubes (Nalgene^®^, VWR International, Bristol, UK), were placed into a freezing container ‘MrFrosty’ (Nalgene^®^) and kept overnight at −80°C before being transferred to −150°C for long-term storage.

Cryopreserved PBMC samples were thawed in a 37°C water bath by gentle continuous mixing. The cell suspension was resuspended in cell medium and thereafter washed (PBS supplemented with 2% FCS) and finally resuspended in RPMI 1640 medium supplemented with 2% FCS.

Cell viability of all PBMC samples was determined with the TC20 automated cell counter (Bio-Rad Laboratories, Hercules, CA, USA) via trypan blue exclusion. The mean cell viability was ≥90% in all samples. All PBMC samples were allowed to rest at room temperature for 1 hour before staining with fluorochrome-conjugated monoclonal antibodies in advance of flow cytometric analysis.

### Flow cytometry and analysis of B cell subsets

#### Staining of PBMC and data collection

All flow cytometric analyses were performed on a BD FACSCanto II Flow cytometer (BD Biosciences, San Jose, CA) using BD FACSDiva software version 8.0 (BD Biosciences) and analyzed by the same person to avoid inter-assay variation. Monoclonal antibodies (mAb) purchased from BD Biosciences were used for the determination of different B cell subset distributions and frequencies in PBMCs. The combination of antibodies used for extracellular surface markers of B cell subsets in the different tubes is presented in Supplementary Table S1. Titrated amounts of fluorochrome-conjugated monoclonal antibodies were added to 5 × 10^5^ PBMC and incubated for 30 minutes at room temperature in darkness. Directly after incubation, cells were washed in 1 mL PBS and resuspended in 500 μl of PBS. Acquisition of data on the flow cytometer was performed within 2 hours after staining. Based on morphological characteristics, acquisition gates were restricted to lymphocyte gates, and a minimum of 150 000 lymphocytes were acquired and analyzed. The results are presented as percentages of the B cell population expressed marker or percentages of B cell population subsets (%) [33].

#### Gating strategy and analysis of data

 Lymphocytes were identified based on their size in the forward scatter (FSC) and morphological characteristics on the side scatter (SSC; Supplementary Figure S1A). Doublet cells and debris were excluded from the analysis using the FSC and SSC areas (widths and heights). Fixable Viability Stain 450 was used to discriminate viable from non-viable cells.

B cells, defined as CD19^+^ lymphocytes (Supplementary Figure S1B), were analyzed for expression of the following differentiation/maturation markers, expressed as % of CD19^+^ cells: CD10, CD24, CD38, CD27, and IgD (Supplementary Figure S1C–S1G); and regulation/activation markers: CD1d, CD5, CD25, CD39, CD95, and CCR7 (Supplementary Figure S1H–S1M).

Based on differentiation/maturation markers the major developmental peripheral B cell subsets were gated as follows: transitional B-cells (CD10^+^CD19^+^, CD24^hi^CD38^hi^CD19^+^, IgD^+^CD10^+^CD24^hi^CD38^hi^CD19^+^), naive B cells (CD27^−^CD19^+^ or IgD^+^CD19^+^), and memory B cells (CD27^+^CD19^+^ or IgD^−^CD19^+^). The percentages of naive and memory subsets expressing the regulation/activation markers (CD1d, CD5, CD39, and CCR7) were determined. Breg cells were classified and gated as CD24^hi^CD38^hi^CD19^+^ (tBreg), CD24^hi^CD27^+^CD19^+^ (mBreg), CD1d^+^CD27^+^CD19^+^, and CD5^+^CD1d^+^CD19^+^ (Supplementary Figure S2A–S2I). Further, the expression of the regulation/activation markers (CD1d, CD5, CD95, and CCR7) was examined on CD24^hi^CD38^hi^CD19^+^ Bregs. The gating strategies are presented in Supplementary Figures S1 and S2.

#### Quality control

The instrument’s performance was checked daily using the set-up and tracking application BD FACS 7-Color Set-up Beads (BD Biosciences). The same lots of mAb were used throughout the study. Compensation beads (BD Biosciences) were used to optimize fluorescence compensation settings for multicolor flow cytometric analysis. Fluorescence minus one (FMO) control (samples that contain all the antibodies in a panel, minus one of them while the others remain constant) was used to ascertain that no false-positive staining occurred. FMOs were used to ensure proper gate settings for all surface markers [34].

All antibodies were titrated to achieve the highest optical signals (mean fluorescence intensity, MFI) for the positive population and the lowest signal for the negative population, representing the optimal signal-to-noise ratio [34].

### Statistical analysis

All statistical analyses were conducted with IBM SPSS Statistics for Windows, Version 25.0. (Armonk, NY: IBM Corp, USA) and GraphPad Prism version 6.0 for (GraphPad, San Diego, CA). Since the sample size was small and the results did not follow a Gaussian distribution, nonparametric tests were used to compare different groups. Mann–Whitney *U*-test was used to compare the different diagnosis groups. Differences were considered statistically significant at *P* < 0.05. All data are presented as median, minimum (min), and maximum (max) values.

## Results

The phenotypical analysis found no differences between the different study groups in percentages of total CD19^+^ B cells expressing the surface markers CD10, CD24, CD38, CD1d, CD25, or CCR7 (Table 2). In contrast, differences were detected in the percentage of both naive (CD27^−^CD19^+^ and IgD^+^CD19^+^) and memory (CD27^+^CD19^+^ and IgD^−^CD19^+^) cells as well as CD5, CD39, and CD95 expressing B cells between the different study groups (Table 2). In more detail, an increased percentage of CD5^+^CD19^+^ cells was observed in children diagnosed exclusively with T1D in comparison with the other study groups (T1D and C: *P* = 0.040, C: *P* = 0.030, and references: *P* = 0.014). Exclusively, children with C had a higher percentage of CD39^+^CD19^+^ cells compared to the reference group (*P* = 0.013). Further, children with a double diagnosis had a higher percentage of CD95^+^CD19^+^ cells compared to T1D (*P* = 0.024). Children with either T1D (*P* = 0.011) or C (*P* = 0.047) showed a lower percentage of CD95^+^CD19^+^ relative to the reference group.

**Table 2.** Distribution of B cell subsets (percentage) in children with T1D + C (*n* = 9), T1D (*n* = 9), C (*n* = 9), and Ref (*n* = 9), presented as median (IQR/range).

	T1D + C	T1D	C	Ref
% CD19^+^ of lymphocytes	9.3 (4.9/10.9)	9.3 (3.9/8.6)	9.5 (4.0/8.7)	9.2 (9.2/19.3)
Differentiation/maturation B cell markers (% of CD19^+^ cells)				
% CD10^+^	16.5 (12.3/27.4)	21.4 (6.7/22.0)	19.3 (11.6/24.2)	24.6 (9.9/16.6)
% CD24^+^	83.2 (6.5/16.0)	81.5 (7.1/18.7)	79.0 (11.8/22.6)	84.7 (10.5/22.2)
% CD38^+^	48.1 (14.1/17.9)	54.1 (10.4/26.8)	52.8 (14.1/18.6)	52.9 (9.3/19.1)
% CD27^−^ (Naive B cells)	81.6 (13.1/34.5) ↓ ^a^^,^^b^	85.5 (6.0/7.3) ↑ ^c^	87.3 (3.8/7.9) ↑ ^c^	81.1 (6.6/17.2)
% IgD^+^ (Naive B cells)	78.9 (10.6/24.9) ↓ ^b^	83.3 (6.0/10.3) ↑ ^c^	87.6 (6.0/10.8) ↑ ^c^	77.3 (8.0/15.3)
% CD27^+^ (Memory B cells)	18.4 (13.1/34.4) ↑ ^a^^,^^b^	14.5 (6.1/7.4) ↓ ^c^	12.8 (3.8/7.8) ↓ ^c^	18.9 (6.5/17.2)
% IgD^−^ (Memory B cells)	21.2 (10.7/25.0) ↑ ^b^	16.8 (5.9/10.4) ↓ ^c^	12.5 (5.9/10.8) ↓ ^c^	22.7 (8.0/15.3)
Regulation/activation B cell markers (% of CD19^+^ cells)				
% CD1d^+^	67.9 (15.0/32.3)	65.0 (10.3/17.4)	71.8 (14.2/22.7)	66.1 (15.2/26.1)
% CD5^+^	9.0 (8.5/14.6) ↓ ^a^	15.6 (12.0/33.8) ↑ ^b^^,^^c^	6.8 (8.5/18.6)	6.8 (5.4/15.8)
% CD25^+^	10.0 (3.8/7.4)	9.7 (3.4/8.8)	11.4 (4.9/7.0)	9.1 (1.6/8.1)
% CD39^+^	51.4 (14.6/26.1)	56.0 (9.7/21.8)	63.0 (10.4/24.6) ↑ ^c^	53.0 (11.0/22.5)
% CD95^+^	12.7 (6.0/13.4) ↑ ^a^	7.3 (3.0/5.8) ↓ ^c^	7.4 (6.4/9.8) ↓ ^c^	12.8 (4.1/16.7)
% CCR7^+^	64.9 (11.6/22.5)	67.3 (12.0/22.3)	68.0 (6.7/14.1)	71.2 (7.5/11.9)

T1D + C, combined type 1 diabetes and celiac disease; T1D, children with type 1 diabetes; C, children with celiac disease; Ref, reference children.

^a^Significant difference (*P* < 0.05) in comparison with T1D.

^b^Significant difference (*P* < 0.05) in comparison with C.

^c^Significant difference (*P* < 0.05) in comparison with Ref. children.

### Differences in naive B cells and their subsets

**Figure 1.**
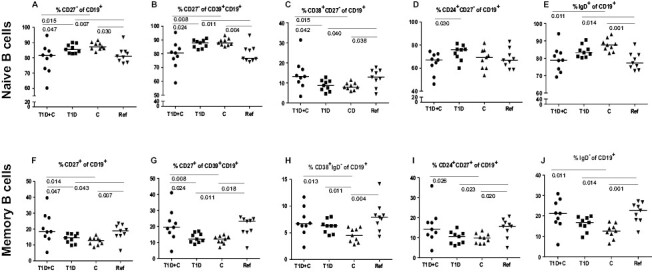
The distribution and differences in percentages (median) of the naive (**A**–**E**) and memory (**F**–**J**) B cells in children with combined type 1 diabetes and celiac disease (T1D + C), type 1 diabetes (T1D), celiac disease (C), and reference children (Ref).

Children with a single diagnosis were characterized by a higher percentage of CD27^−^CD19^+^ (T1D: *P* = 0.047, C: *P* = 0.015, Fig. 1A) and CD39^+^ expressing naive B cells (T1D: *P* = 0.024, C: *P* = 0.008, Fig. 1B); but lower percentage of CD38^+^ expressing naive B cells (T1D: *P* = 0.042, C: *P* = 0.015, Fig. 1C) compared to children with double diagnosis. Similarly, children with T1D or C showed an increased percentage of CD27^−^CD19^+^ (T1D: *P* = 0.007; C: *P* = 0.030, Fig. 1A) and CD39^+^ expressing naive B cells (T1D: *P* = 0.007, C: *P* = 0.030, Fig. 1B); but a lower percentage of CD38^+^ expressing naive B cells (T1D: *P* = 0.040, C: *P* = 0.038, Fig. 1C) in comparison with the reference children.

Children with T1D exclusively had an increased percentage of CD24^+^CD27^−^ naive B cells compared to children in the double diagnosis group (*P* = 0.030, Fig. 1D).

The percentages of IgD^+^CD19^+^ cells were lower in the double diagnosis group compared to children with C (*P* = 0.011, Fig. 1E). Contrary, the percentages of IgD^+^ naive B cells were increased in children diagnosed exclusively with T1D or C in comparison with reference children (T1D: *P* = 0.014, C: *P* = 0.001, Fig. 1E).

### Differences in memory B cells and their subsets

In the double diagnosis group, percentages of CD27^+^CD19^+^ memory B cells (Fig. 1F) were higher compared to T1D (*P* = 0.047) and C (*P* = 0.014) children. Children with T1D (*P* = 0.043) or C (*P* = 0.007) had a lower percentage of CD27^+^CD19^+^ memory B cells in comparison with reference children (Fig. 1F). Analysis of activation associated CD39^+^ surface marker on CD27^+^ memory B cells found lower percentages of CD39^+^CD27^+^ cells in the T1D group (Fig. 1G) compared to the double diagnosis group (*P* = 0.024) or reference children (*P* = 0.011). Similarly, children diagnosed with C had a lower percentage of CD39^+^CD27^+^ B cells compared to the double diagnosis group (*P* = 0.008) and reference children (*P* = 0.018; Fig. 1G).

The percentages of CD38^+^IgD^−^ B cells in children diagnosed exclusively with C were lower compared to the other study groups (T1D and C: *P* = 0.013; T1D: *P* = 0.011, reference: *P* = 0.004, Fig. 1H). The percentages of CD24^+^CD27^+^ cells were also lower in children with C compared to the double diagnosis group (*P* = 0.026) and reference children (*P* = 0.020), respectively (Fig. 1I).

The percentages of IgD^+^CD19^+^ cells were higher in the double diagnosis group compared to children with C (*P* = 0.011, Fig. 1J). Contrary, percentages of IgD^−^ memory B cells were lower in children diagnosed exclusively with T1D or C compared to reference children (T1D: *P* = 0.014, C: *P* = 0.001, Fig. 1J).

The percentages of CCR7^+^IgD^−^ B cells were lower in children diagnosed with T1D (*P* = 0.013) or C (*P* = 0.003) compared to reference children (data not shown). Further, children with a double diagnosis had a higher percentage of CCR7^+^IgD^−^ relative to celiac disease-diagnosed children (*P* = 0.032).

### Breg subsets

**Figure 2.**
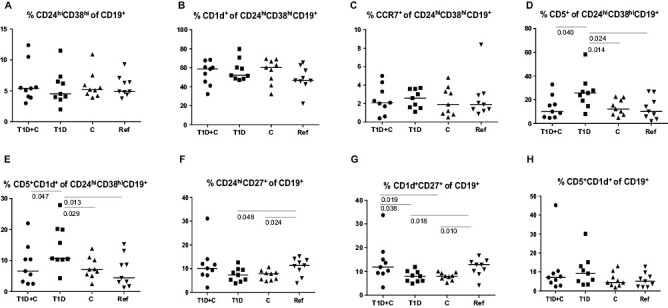
The distribution and differences in percentages (median) of Bregs in children with combined type 1 diabetes and celiac disease (T1D + C), type 1 diabetes (T1D), celiac disease (C), and reference children (Ref).

Among B cells with regulatory capacity, no differences were observed in percentages of tBreg (CD24^hi^CD38^hi^CD19^+^) and CD1d^+^ or CCR7 expressing tBreg between the different study groups (Fig. 2A–C). Exclusively type 1 diabetic children had an increased percentage of CD5 expressing CD24^hi^CD38^hi^CD19^+^ Bregs in comparison with the other study groups (T1D and C: *P* = 0.040, C: *P* = 0.014, and reference: *P* = 0.024, Fig. 2D). Similarly, children diagnosed with T1D had a higher percentage of CD5^+^CD1d^+^ expressing CD24^hi^CD38^hi^CD19^+^ Bregs compared to other study groups (T1D and C: *P* = 0.047, C: *P* = 0.029, and reference: *P* = 0.013, Fig. 2E).

Further, mBreg (CD24^hi^CD27^+^) percentages were decreased in the single diagnosis groups (T1D: *P* = 0.048, C: *P* = 0.024, Fig. 2F) compared to the reference group. Similarly, children with a single diagnosis had lower percentages of CD1d^+^CD27^+^ Bregs than children with a double diagnosis (T1D: *P* = 0.038, C: *P* = 0.019) or reference children (T1D: *P* = 0.018, C: *P* = 0.010; Fig. 2G). No differences were found in percentages of CD5^+^CD1d^+^CD19^+^ Bregs between the different study groups (Fig. 2H).

## Discussion

Increasing evidence suggests that B cells are as important as T cells in the immunopathogenesis of autoimmune diseases and play both positive effector and negative regulatory roles during immune responses [1]. The current study thus aimed to gain a deeper understanding of B cell subset patterns involved in T1D and C pathophysiology in children. We hypothesized that alterations in the phenotypical pattern of peripheral B cell subsets might occur between children with single or double diagnoses of the autoimmune disease T1D and C, respectively.

**Figure 3.**
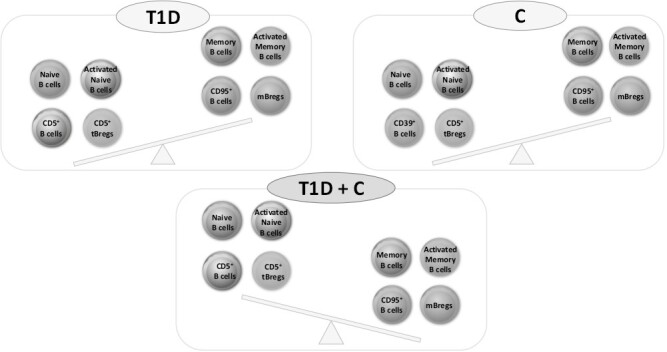
Alterations in the different B subsets in children with combined type 1 diabetes and celiac disease (T1D + C), type 1 diabetes (T1D), celiac disease (C), and reference children (Ref).

The major findings, summarized in Fig. 3, indicate a distinct alteration in B cell subsets, including naive, memory, and Breg cells, across the different diagnosis groups. The most striking difference was observed between children with a combined diagnosis of T1D and C compared to children with exclusively one of these diseases. Naive B cells constitute about 70–80% of circulating B cells, preferentially producing the anti-inflammatory cytokine IL-10 and consisting of cells at different maturation stages (immature and naive mature B cells) and with heterogeneous characteristics [3, 6]. Based on the expression of single differentiation/maturation markers, the phenotyping results demonstrated significant differences in the percentage of naive and memory B cells between children with autoimmune diseases and reference children. Our results regarding both naïve (Md 81.1%) and memory (Md 18.9%) B cells in our group of reference children are in line with age-adjusted reference values recommended by Duchamp et al., i.e naïve (Md 74.4%) and memory (Md 17.4%) B cells, respectively. Similarly, both the percentage of B cells (CD19^+^, Md 9.2%) and CD24^hi^CD38^hi^ Breg cells (Md 5.8%) in the reference group is in line with previous studies [6, 7].

Both children with T1D and C had an increased percentage of naive B cells and lower percentages of memory cells. In agreement with our data, increased frequencies of naive/immature B cells have previously been reported in the context of autoimmune diseases [5, 23, 28]. Wardemann et al. [35] showed that a majority (55–75%) of all antibodies expressed by naive B cells displayed self-reactivity. This leads us to speculate that the increased frequency of naive B cells can reflect the increased generation of autoantibodies seen in autoimmune diseases. However, in the context of combined diagnoses, this does not seem to be coherent. Our data indicated a lower frequency of naive (CD27^−^CD19^−^ and IgD^+^CD19^+^) B cells in children with combined diagnosis of T1D and C. Since the proportion of total B cells in the different diagnosis groups was comparable with the reference group, the altered frequency of naive cells presumably depends on a repertoire shifting between the different subsets. However, these B cells are an important contributor to the generation of autoantibodies even if, in multiple autoimmune diseases, disturbances at later stages of B cell differentiation seem to be of more importance.

The percentage of CD39^+^ expressing CD27^−^ naive B cells varied across groups. Upregulation of CD39 on the B cell surface is associated with activation and an enhanced capacity to suppress the T-effector cells [21]. When T1D and C coexist, the frequency of CD39^+^CD27^−^ activated B cells was lower compared to the single diagnosis groups. This may indicate an additional disturbance in B cell-mediated suppression of T-cell response in children with both T1D and C. Speculatively, this may contribute to the development of a second autoimmune disease in the child.

Similar to naive B cells, divergent results regarding frequencies of circulating memory B cells and mBregs in autoimmune diseases are reported [8, 24, 36]. We can here show a lower frequency of memory B cells defined as CD27^+^ or IgD^−^ coherent with percentages of CD95^+^ expressing B cells in children with T1D, as reported by others [8, 24]. Contrary, we found increased percentages of memory B cells and activated B memory cells (CD39^+^CD27^+^) in children with combined diagnosis. The enhanced immune response and B cell activation (increased CD39^+^ expression) is probably one mechanism that contributes to the expansion of memory B cells in the peripheral blood of children with combined T1D and C. So far, no other studies have reported results regarding CD38 expression on B cells in the context of celiac disease. Studies have shown that gluten challenge induces the expression of CD38^+^ on T cells [37, 38]. We speculate that the lower percentage of CD38^+^ memory B cells seen in children with C in comparison to the other study groups partly can depend on the discrepancy in gluten challenge ‘status’ and celiac disease duration.

Regulatory B cells are challenging to study because of the lack of a specific Breg-associated marker [9, 11, 14, 18]. The importance of CD1d expression in the regulatory function of B cells has been demonstrated in the context of chronic intestinal inflammatory disease [30] and systemic lupus erythematosus [16]. B cells expressing CD1d are partly involved in autoimmunity regulation as a checkpoint for B cell activation with subsequent auto-antibody production and contributions to disease progression [39]. Deng et al. have described a lower frequency of CD1d expressing B cells in T1D patients, in agreement with our findings in children with T1D or C. The increased frequency of CD1d^+^CD27^+^CD19^+^ (mBregs) in children with a double diagnosis, compared to a single diagnosis, can be explained by the fact that checkpoint of activation and subsequent auto-antibody production [39] are partly involved in the enhanced auto-antibody secretion, thereby exacerbating autoimmune processes.

The CD5^+^ B cells’ capacity to downregulate autoimmune inflammation and maintain tolerance by IL-10 secretion was previously demonstrated [17, 40, 41]. Our results of an increased percentage of CD5^+^ B cells in T1D children correlate well with earlier studies [42, 43]. These studies also indicate the importance of CD5^+^ B cells in the pathogenesis of T1D. Exclusively children with T1D had increased percentages of CD5^+^ B cells, CD5^+^ of CD24^hi^CD38^hi^CD19^+^ (tBreg), and CD5^+^CD1d^+^ of CD24^hi^CD38^hi^CD19^+^ expressing Bregs in comparison with the other studied groups. We speculated that the higher frequency of CD5^+^ expressing B subsets in the diabetes group could be associated with the anti-inflammatory capacity of CD5^+^ B cells. Possibly, this can act protectively against the development of C in children with T1D. However, further studies are needed to elucidate the functional role of CD5^+^ expressing B cells in T1D, as well as the possibility of using it as a predictive marker in the development of C in children already diagnosed with T1D.

Previous studies reported divergent results regarding CD24^hi^CD38^hi^CD19^+^ and CD24^hi^CD27^+^CD19^+^ Bregs in T1D [28, 36, 44, 45]. Our results showed a lower percentage of CD24^hi^CD27^+^CD19^+^ Bregs and also lower (but not significant) percentages of CD24^hi^CD38^hi^CD19^+^ in children with T1D in line with results reported by Wang et al. [44] and El-Mokhtar et al. [45]. In the C group, we found lower percentages of CD24^hi^CD27^+^CD19^+^ Bregs, contrary to the previously reported increased (but not significant) percentage of Bregs in adults [29]. The divergent results could depend on differences in age, disease duration, and time of gluten-free diet in the studied groups. Interestingly, in our study, the percentages of Bregs with a memory phenotype (CD24^hi^CD27^+^CD19^+^ and CD1d^+^CD27^+^CD19^+^) in children with a double diagnosis were increased in comparison with those having a single diagnosis. The *in vivo* mechanisms of action of CD24^hi^CD38^hi^CD19^+^ and CD24^hi^CD27^+^CD19^+^ Bregs are not the same [41]. CD24^hi^CD27^+^CD19^+^ are more efficient than CD24^hi^CD38^hi^ in the suppression of CD4^+^ T-cell proliferation, IFN-γ and IL-17 production from monocytes and CD4^+^ T cells, and play an exclusive role in the induction of IL-10^+^ T cells [15]. The lower percentages of mBregs can influence B cell mediated suppression, contributing to diminished induction of IL-10^+^ T cells in children with T1D or C. Thereby, this could possibly be a contributing factor to the development of another autoimmune disease. However, further studies are needed to understand the reason why the tBregs are lower and mBreg subsets are increased in children with double diagnosis, as this indicates an opposite effect of different Bregs in case of coexistence of these autoimmune disorders.

Regarding limitations, our study has both strengths and some weaknesses. The main strength of the study is the wide range of studied B subsets in a clinical context of children with T1D and/or C. Another important strength of this study is that the results on frequencies of the major B cell subsets in the reference groups are consistent with reference values recommended by previous studies [6, 7]. One limitation of our study is the relatively small sample size in the different diagnosis groups and the heterogeneity in the duration of diseases among the participants. However, we suppose that this does not reduce the significance of the found differences between the groups because we did not find significant differences regarding age, duration of disease, and percentages of total B cells between the study groups. This gives evidence that our cohort is representative even if the sample size is relatively small.

In summary, a larger compartment of naive B and Breg subsets, including activated subsets, and smaller compartment of the memory B and Breg subsets are seen in children with isolated T1D or C. In children with the combined diagnosis, a shifting of the naive B subset compartment toward a more mature/differentiated B cell compartment was observed, indicating another type of disturbance of the B cell mediated immune regulation compared to what is present in the context of only one isolated autoimmune disease. Following these results, further evaluation of the involved B subsets, specific surface markers, plasticity, and their respective functionality is necessary using larger cohorts. This will allow for an appraisal of the clinical relevance of these findings.

## Supplementary Material

uxad136_suppl_Supplementary_Table

uxad136_suppl_Supplementary_Figure_S1

uxad136_suppl_Supplementary_Figure_S2

## Data Availability

The data underlying this article will be shared upon reasonable request to the corresponding author.

## Abbreviations

C: celiac disease
CD: cluster of differentiation
FSC: forward scatter
mBreg: memory B regulatory cell
T1D: type 1 diabetes
tBreg: transitional B regulatory cell
