# Supplementary material for: Shift in the B cell subsets between children with type 1 diabetes and/or celiac disease
Source: Clin Exp Immunol. 2023 Dec 22;216(1):36–44. doi: 10.1093/cei/uxad136 (PMC10929695; doi:10.1093/cei/uxad136)
Supplement: uxad136_suppl_Supplementary_Table [file uxad136_suppl_supplementary_table.docx]

**Supplement table**

Monoclonal antibodies used for staining and flow cytometric analysis of B cell subsets.

|  | **Antibody*** | **Fluorochrome** | **Clone** |
| --- | --- | --- | --- |
| **Tube 1** | CD39 | FITC | TU66 |
|  | CD1d | PE | CD1d42 |
|  | CD19 | PerCP-Cy5.5 | HIB19 |
|  | CD24 | PE-Cy7 | ML5 |
|  | CD27 | APC | M-T271 |
|  | CD38 | APC-H7 | HB7 |
|  | FVS450 | BV421 | - |
|  | CD5 | BV510 | UCHT2 |
| **Tube 2** | IgD | BB515 | IA6-2 |
|  | CD10 | PE | HI10a |
|  | CD19 | PerCP-Cy5.5 | HIB19 |
|  | CD25 | PE-Cy7 | M-A251 |
|  | CCR7 (CD197) | Alexa Fluor 647 | 150503 |
|  | CD38 | APC-H7 | HB7 |
|  | CD95 | BV421 | -/ DX2^2^ |
|  | CD24 | BV510 | ML5 |

* All antibodies were manufactured by BD Biosciences.

FITC=fluorescein-isothiocyanate; PE=phycoerythrin; PerCP=peridinin-chlorophyll-protein; Cy=Cyanine; APC=allophycocyanin; BV=Brilliant Violet™; BB=Brilliant™ Blue; CCR7=Chemokine Receptor; FVS450=Fixable Viability Stain 450.
