# Supplementary material for: Shift in the B cell subsets between children with type 1 diabetes and/or celiac disease
Source: Clin Exp Immunol. 2023 Dec 22;216(1):36–44. doi: 10.1093/cei/uxad136 (PMC10929695; doi:10.1093/cei/uxad136)
Supplement: uxad136_suppl_Supplementary_Figure_S1 [file uxad136_suppl_supplementary_figure_s1.pptx]

## Slide 1
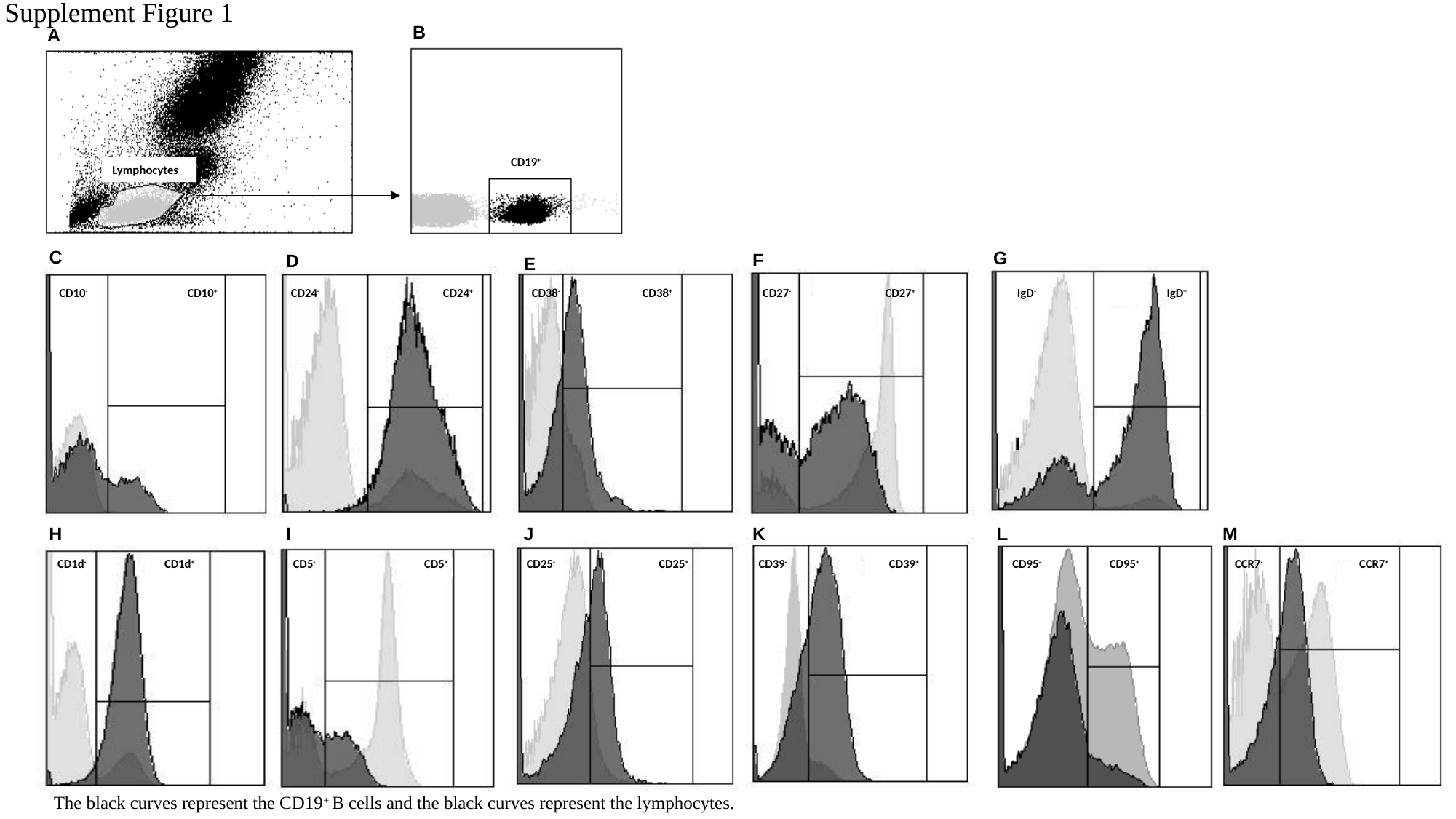

Supplement Figure 1
B
A
CD19+
Lymphocytes
C
G
F
D
E
CD10-
CD10+
CD24-
CD24+
CD38-
CD38+
CD27-
CD27+
IgD-
IgD+
I
H
I
J
K
L
M
CD1d-
CD1d+
CD5-
CD5+
CD25-
CD25+
CD39-
CD39+
CD95-
CD95+
CCR7-
CCR7+
The black curves represent the CD19+ B cells and the black curves represent the lymphocytes.
