# Supplementary material for: Shift in the B cell subsets between children with type 1 diabetes and/or celiac disease
Source: Clin Exp Immunol. 2023 Dec 22;216(1):36–44. doi: 10.1093/cei/uxad136 (PMC10929695; doi:10.1093/cei/uxad136)
Supplement: uxad136_suppl_Supplementary_Figure_S2 [file uxad136_suppl_supplementary_figure_s2.pptx]

## Slide 1
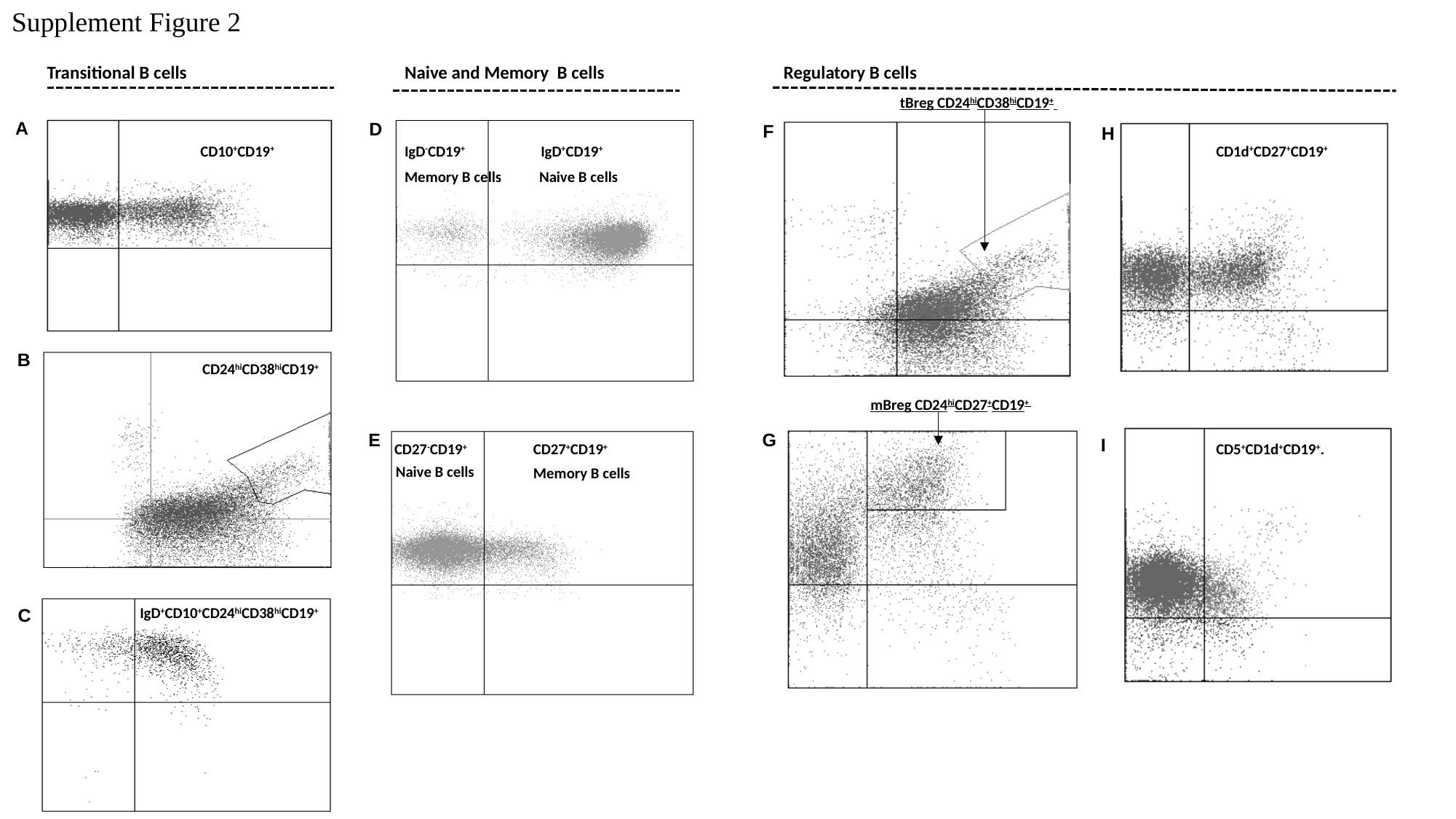

Supplement Figure 2
Transitional B cells
Naive and Memory B cells
Regulatory B cells
tBreg CD24hiCD38hiCD19+
A
D
F
H
CD10+CD19+
IgD-CD19+
IgD+CD19+
CD1d+CD27+CD19+
Memory B cells
Naive B cells
B
CD24hiCD38hiCD19+
mBreg CD24hiCD27+CD19+
E
G
I
CD27-CD19+
CD27+CD19+
CD5+CD1d+CD19+.
Naive B cells
Memory B cells
IgD+CD10+CD24hiCD38hiCD19+
C
